# Gene expression microarray data from mouse cerebrum treated with rTMS for 30 days

**DOI:** 10.1016/j.dib.2017.10.034

**Published:** 2017-10-19

**Authors:** Tetsurou Ikeda, Satoru Kobayashi, Chikao Morimoto

**Affiliations:** a Laboratory for Structural Neuropathology, RIKEN Brain Science Institute, 2-1 Hirosawa, Wako-shi, Saitama 351-0198, Japan; b Clinical Immunology, Institute of Medical Science, The University of Tokyo, 4-6-1 Shirokanedai, Minato-ku, Tokyo 108-8639, Japan; c Technical Support, Thermo Fisher Scientific K.K., 3-9 Moriya, Kanagawa-ku, Yokohama 221-0022, Japan

## Abstract

This data article contains complementary tables related to the research article entitled, ‘Effects of repetitive transcranial magnetic stimulation on ER stress-related genes and glutamate, γ-aminobutyric acid, and glycine transporter genes in mouse brain’ (Ikeda et al. (2017) [1]), which showed that rTMS modulates glutamate, GABA and glycine transporters and regulates ER stress-related genes. Here we provide accompanying data collected using Affymetrix GeneChip microarrays to identify changes in gene expression in mouse cerebrum treated with rTMS for 30 days (Tables 1–10).

**Specifications Table**

**Table:** 

Subject area	*Neuroscience*
More specific subject area	*Psychiatric disorders*
Type of data	*Tables*
How data was acquired	*Affymetrix GeneChip RNA microarray*
Data format	*Filtered, analysed*
Experimental factors	*Mouse brain treated with rTMS for 30 days*
Experimental features	*RNA isolation, global gene expression analyses*
Data source location	*Wako, Saitama, Japan*
Data accessibility	*Data are contained within this article*

**Value of the Data**

• A global gene expression analysis of mouse cerebrum treated with rTMS for 30 days.
• These data may be useful for comparison with microarray data obtained from rTMS of different durations.
• Genes identified as differentially expressed in this data set could be useful in further studies on the effects of rTMS on mouse brain.

## 1. Data

Affymetrix GeneChip microarray analyses of mRNA isolated from mouse cerebrum after 30 days of rTMS revealed altered expression of several genes (Table 1, Table 2, Table 3, Table 4, Table 5, Table 6, Table 7, Table 8, Table 9, Table 10), including glutamatergic genes (e.g., glutamate transporter), dopaminergic genes, cholinergic genes, genes of adrenergic signaling in cardiomyocytes and so on.

**Table 1:** Gene expression matrix after 30 days rTMS on cerebrum.

**TC ID**	**GS**	**L1**	**C**	**L2**	**C**	**L3**	**C**	**L4**	**C**	*	**#**	**Description**
93320_at	Cpt1a	0.44	I	0.15	I	0.3	I	0.17	I	4	0	carnitine palmitoyltransferase 1a, liver
93372_at	Anp32a	1.75	I	2.28	I	1.59	I	1.91	I	4	0	acidic (leucine-rich) nuclear phosphoprotein 32 family, member A
95466_at	Cotl1	2.41	I	1.11	I	2.03	I	0.83	I	4	0	coactosin-like 1 (Dictyostelium)
103012_at	Ccl21a	0.27	I	3.54	I	-1.5	D	1.6	I	3	1	chemokine (C-C motif) ligand 21A (serine); chemokine (C-C motif)
												ligand 21B (leucine); chemokine (C-C motif) ligand 21C (leucine);
												predicted gene 10591; predicted gene 13304; predicted gene 1987;
												predicted gene, 21541; C-C motif chemokine 21c
												
100307_at	Nfix	1.12	I	0.62	I	0.41	I	-0.05	NC	3	0	nuclear factor I/X
101883_s_at	Xlr3a	0.03	NC	1.16	I	0.74	I	1.79	I	3	0	X-linked lymphocyte-regulated 3A; X-linked lymphocyte-regulated
												3B; X-linked lymphocyte-regulated 3C
												
101921_at	Rab4a	0.46	I	0.27	I	-0.01	NC	0.07	I	3	0	RAB4A, member RAS oncogene family
104175_at	Dlg4	1.09	I	1.46	I	0.41	I	1.15	NC	3	0	discs, large homolog 4 (Drosophila)
93253_at	Mapk1	-0.04	NC	0.34	I	0.34	I	0.71	I	3	0	mitogen-activated protein kinase 1
93924_f_at	Tuba3b	0.4	I	0.15	I	0.11	I	-0.09	NC	3	0	tubulin, alpha 3B
96295_at	Psat1	0.09	NC	0.11	I	0.28	I	0.44	I	3	0	phosphoserine aminotransferase 1
96590_f_at	Otud7b	0.59	NC	0.7	I	0.33	I	0.32	I	3	0	OTU domain containing 7B
99598_g_at	Gnai2	0.33	I	0.67	I	-0.1	NC	0.2	MI	3	0	guanine nucleotide binding protein (G protein), alpha inhibiting 2
102009_at	Cyfip2	0.53	I	0.21	I	-0.33	NC	-0.6	D	2	1	cytoplasmic FMR1 interacting protein 2
103275_at	Atp6v0a1	0.52	I	-1.05	D	1.2	I	-0.31	NC	2	1	ATPase, H+ transporting, lysosomal V0 subunit A1
104486_at	A2m	0.27	I	-0.91	D	0.83	I	-0.91	NC	2	1	alpha-2-macroglobulin
104564_at	Scg3	0.47	I	0.32	I	-0.49	D	-0.52	NC	2	1	secretogranin III
104643_at	Wwc1	0.72	I	0.47	I	-0.34	NC	-0.69	D	2	1	WW, C2 and coiled-coil domain containing 1
160189_at	Nudt4	-0.2	NC	-0.84	D	0.63	I	0.4	I	2	1	nudix (nucleoside diphosphate linked moiety X)-type motif 4
162138_s_at	Cbx6	0.21	I	0.27	I	-0.54	D	-0.71	NC	2	1	chromobox 6
93660_at	Camk2a	1.53	I	-0.93	D	2.09	I	-0.27	NC	2	1	calcium/calmodulin-dependent protein kinase II alpha
95301_at	S100a5	0.6	I	0.96	I	-0.86	D	-0.45	NC	2	1	S100 calcium binding protein A5
95785_s_at	Rab7	-0.36	NC	-0.91	D	0.91	I	0.4	I	2	1	RAB7, member RAS oncogene family
96583_s_at	Kif5a	-0.13	NC	-1.32	D	1.6	I	0.36	I	2	1	kinesin family member 5A
97458_at	Gnb1	0.61	I	0.8	I	-0.58	D	-0.62	NC	2	1	guanine nucleotide binding protein (G protein), beta 1
97560_at	Psap	-0.28	NC	-1.49	D	1.74	I	0.37	I	2	1	prosaposin
98457_at	Slc4a4	0.72	I	0.35	I	-0.62	NC	-0.92	D	2	1	solute carrier family 4 (anion exchanger), member 4
99458_i_at	Mark2	0.28	I	0.46	I	-0.66	D	-0.5	NC	2	1	MAP/microtubule affinity regulating kinase 2
99481_at	Atp1a2	0.86	I	0.45	I	-0.49	NC	-0.82	D	2	1	ATPase, Na+/K+ transporting, alpha 2 polypeptide
99882_at	Ids	-0.75	NC	-1.24	D	0.55	I	-0.19	MI	2	1	iduronate 2-sulfatase
100012_at	Laptm5	0.63	I	0.07	NC	0.34	MI	-0.35	NC	2	0	lysosomal-associated protein transmembrane 5
100068_at	Aldh1a1	0.21	I	0.16	I	-0.42	NC	-0.31	NC	2	0	aldehyde dehydrogenase family 1, subfamily A1
100133_at	Fyn	0.35	I	0.55	I	-0.22	NC	0.06	NC	2	0	Fyn proto-oncogene
100154_at	Tapbp	0.8	I	0.21	I	0.28	NC	-0.19	NC	2	0	TAP binding protein
100380_at	Gm10257	0.06	NC	0.26	I	-0.21	NC	0.02	I	2	0	predicted gene 10257; predicted gene 12657; H3 histone, family 3A;
												H3 histone, family 3B; H3 histone, family 3C; histone H3.3-like;
												uncharacterized LOC105242736
100494_at	Fgf1	0.48	NC	-0.23	NC	0.71	I	0.29	I	2	0	fibroblast growth factor 1
100573_f_at	Gpi1	0.35	I	0.41	I	-0.13	NC	-0.05	NC	2	0	glucose phosphate isomerase 1
100727_at	Rpl28	0.36	I	0.6	I	-0.26	NC	-0.02	NC	2	0	ribosomal protein L28
100762_at	Sema6a	2.15	I	-0.09	NC	0.39	MI	-1.7	NC	2	0	sema domain, transmembrane domain (TM), and cytoplasmic
												domain, (semaphorin) 6A

Gnb1, Gnai2, Dlg4 and Mapk1 are glutamatergic genes. Fyn, Camk2a and Mapk1 are cholinergic genes. Kif5a is Dopaminergic gene. Mice cerebrum stimulated by rTMS for 30days were denoted as M1 and M2, sham control were denoted as C1 and C2. N=2. All expression ratios were converted into the log_2_(expression ratio) values. L1: Signal Log Ratio (M1/C1), L2: Signal Log Ratio (M2/C1), L3: Signal Log Ratio (M1/C2), L4: Signal Log Ratio (M1/C2). C1, C2 were used as a control and M1, M2 were normalized with respect to C1 and C2 to obtain expression ratios. Expression Console (EC) Ver.1.4 and Transcriptome Analysis Console (TAC) Ver.3. were used for Comparison Analysis as manufacture procedure. C means data analysis output for a Comparison Analysis illustrating Change p-values with the associated Increase (I) or Decrease (D) call. Increase calls have Change p-values closer to zero and Decrease calls have Change p-values closer to one. Finally, the Change algorithm assesses probe pair saturation, calculates a Change p-values, and assigns an (I), Marginal Increase (MI), No Change (NC), Marginal Decrease (MD), or (D) call for C. Gene with more than 2 significant difference calls was chosen. Abbreviations; TC ID: Transcript Cluster ID, GS: Gene Symbol, *: Total number of increase, #: Total number of decrease. TC ID is available for Pathway analysis.

**Table 2:** Gene expression matrix after 30 days rTMS on cerebrum.

**TC ID**	**GS**	**L1**	**C**	**L2**	**C**	**L3**	**C**	**L4**	**C**	*^​^	**#**	**Description**
100774_at	Synj2bp	0.34	I	-0.15	I	-0.28	NC	-0.08	NC	2	0	synaptojanin 2 binding protein
100892_at	Ndufaf1	0.4	NC	0.31	I	0.26	NC	0.29	I	2	0	NADH dehydrogenase (ubiquinone) 1 alpha subcomplex, assembly
												factor 1
100992_at	Phc1	0.43	I	0.24	I	-0.07	NC	-0.28	NC	2	0	polyhomeotic-like 1 (Drosophila)
101113_at	Rhoa	0.31	I	0.16	MI	0.18	NC	-0.26	NC	2	0	ras homolog gene family, member A
101419_at	Tubb4a	0.35	I	0.51	I	-0.27	NC	-0.01	NC	2	0	tubulin, beta 4 A class IVA
101441_i_at	Itpr2	0.32	I	0.25	MI	-0.18	NC	-0.57	NC	2	0	inositol 1,4,5-triphosphate receptor 2
101467_at	S100b	0.06	NC	0.04	NC	0.53	I	0.44	I	2	0	S100 protein, beta polypeptide, neural
101510_at	Psme1	0.21	NC	0.27	I	0.08	NC	0.19	I	2	0	proteasome (prosome, macropain) activator subunit 1 (PA28 alpha)
101578_f_at	Actb	0.92	I	0.99	I	-0.4	NC	-0.47	NC	2	0	actin, beta
101587_at	Ephx1	0.9	I	0.99	I	-1.13	NC	-1.27	NC	2	0	epoxide hydrolase 1, microsomal
101855_at	Map6	0.3	I	0.15	I	-0.01	NC	-0.35	NC	2	0	microtubule-associated protein 6
101923_at	Pla2g7	0.47	I	0.42	I	-0.08	NC	-0.18	NC	2	0	phospholipase A2, group VII (platelet-activating factor
												acetylhydrolase, plasma)
101930_at	Nfix	0.63	I	0.34	MI	-0.11	NC	-0.34	NC	2	0	nuclear factor I/X
101960_at	Rtcb	0.24	NC	0.3	I	-0.18	NC	0.04	I	2	0	RNA 2',3'-cyclic phosphate and 5'-OH ligase
102007_at	Hccs	0.02	NC	-0.35	NC	0.41	I	0.48	I	2	0	holocytochrome c synthetase
102033_at	Tesk1	0.42	I	0.4	I	0.09	NC	-0.05	NC	2	0	testis specific protein kinase 1
102063_at	Pdpk1	0.87	I	0.2	I	-0.2	NC	-0.46	NC	2	0	3-phosphoinositide dependent protein kinase 1
102095_f_at	Spock2	0.32	I	0.18	MI	-0.53	NC	-0.73	NC	2	0	sparc/osteonectin, cwcv and kazal-like domains proteoglycan 2
102252_at	Pfdn2	0.42	I	0.24	I	-0.03	NC	-0.14	NC	2	0	prefoldin 2
102271_at	Zmiz2	0.58	I	0.54	I	-0.32	NC	-0.52	NC	2	0	zinc finger, MIZ-type containing 2
102374_at	Rcan3	0.27	NC	-0.03	NC	0.29	I	0.12	I	2	0	regulator of calcineurin 3
102384_at	Smarca2	0.42	I	0.59	I	-0.31	NC	-0.04	NC	2	0	SWI/SNF related, matrix associated, actin dependent regulator of
												chromatin, subfamily a, member 2
102639_at	Chst2	0.47	I	0.4	I	-0.04	NC	-0.21	NC	2	0	carbohydrate sulfotransferase 2
102691_at	Zfp385a	0.88	I	0.67	I	-0.18	NC	-0.56	NC	2	0	zinc finger protein 385A
102700_at	Tbr1	0.43	I	0.41	I	-0.37	NC	-0.43	NC	2	0	T-box brain gene 1
102752_at	Cyfip1	0.43	I	0.31	I	-0.17	NC	-0.32	NC	2	0	cytoplasmic FMR1 interacting protein 1
102787_at	Adgrg1	0.79	I	0.26	I	0.52	NC	-0.42	NC	2	0	adhesion G protein-coupled receptor G1
102815_at	Anxa11	0.28	I	0.5	I	-0.86	NC	-0.55	NC	2	0	annexin A11; predicted gene 2260; predicted gene 2274
102856_at	Sox10	0.39	I	0.45	I	-0.08	NC	-0.14	NC	2	0	SRY (sex determining region Y)-box 10
102912_at	Tnks2	0.28	I	0.34	I	-0.4	NC	-0.2	NC	2	0	tankyrase, TRF1-interacting ankyrin-related ADP-ribose
												polymerase 2
102942_at	Specc1	0.3	I	0.28	I	-0.35	NC	-0.43	NC	2	0	sperm antigen with calponin homology and coiled-coil domains 1
103001_at	Vegfb	0.46	MI	0.15	I	-0.13	NC	-0.02	NC	2	0	vascular endothelial growth factor B
103029_at	Pdcd4	0.76	I	0.61	I	-0.17	NC	-0.02	NC	2	0	programmed cell death 4
103040_at	Cd83	0.02	NC	0.43	I	-0.12	NC	0.11	I	2	0	CD83 antigen
103054_at	Polr2a	0.43	I	0.07	NC	0.23	I	-0.06	NC	2	0	polymerase (RNA) II (DNA directed) polypeptide A
103090_at	Uqcc1	-0.41	NC	-0.09	NC	0.21	I	0.33	I	2	0	ubiquinol-cytochrome c reductase complex assembly factor 1
103299_at	Pld4	0.88	I	0.03	NC	1.18	I	0.36	NC	2	0	phospholipase D family, member 4
103300_at	Abcb7	0.64	I	-0.12	NC	0.41	I	-0.48	NC	2	0	ATP-binding cassette, sub-family B (MDR/TAP), member 7
103305_at	Itgb4	0.83	I	2.63	I	0.51	NC	2.83	NC	2	0	integrin beta 4
103369_at	Klf13	0.77	I	0.36	I	-0.36	NC	-0.43	NC	2	0	Kruppel-like factor 13
103370_at	Lin7c	0.35	I	0.27	I	0	NC	-0.21	NC	2	0	lin-7 homolog C (C. elegans)
103404_at	Rere	0.6	I	0.19	I	0.05	NC	-0.15	NC	2	0	arginine glutamic acid dipeptide (RE) repeats
103411_at	Gna11	0.23	NC	0.48	I	0.1	NC	0.13	I	2	0	guanine nucleotide binding protein, alpha 11
103584_at	Cmip	0.32	I	0.46	I	-0.37	NC	-0.37	NC	2	0	c-Maf inducing protein

Actb, Itpr2 and Rho are oxytocin signaling pathway genes. Cyfip1and Itgb4 are actin cytoskeleton regulation genes. Pdpk1 and Pdcd4 are genes of proteoglycans in cancer. Pla2g7 amd Pld4 are ether lipid metabolism genes. Mice cerebrum stimulated by rTMS for 30days were denoted as M1 and M2, sham control were denoted as C1 and C2. N=2. All expression ratios were converted into the log_2_(expression ratio) values. L1: Signal Log Ratio (M1/C1), L2: Signal Log Ratio (M2/C1), L3: Signal Log Ratio (M1/C2), L4: Signal Log Ratio (M1/C2). C1, C2 were used as a control and M1, M2 were normalized with respect to C1 and C2 to obtain expression ratios. Expression Console (EC) Ver.1.4 and Transcriptome Analysis Console (TAC) Ver.3. were used for Comparison Analysis as manufacture procedure. C means data analysis output for a Comparison Analysis illustrating Change p-values with the associated Increase (I) or Decrease (D) call. Increase calls have Change p-values closer to zero and Decrease calls have Change p-values closer to one. Finally, the Change algorithm assesses probe pair saturation, calculates a Change p-values, and assigns an (I), Marginal Increase (MI), No Change (NC), Marginal Decrease (MD), or (D) call for C. Gene with more than 2 significant difference calls was chosen. Abbreviations; TC ID: Transcript Cluster ID, GS: Gene Symbol, *: Total number of increase, #: Total number of decrease. TC ID is available for Pathway analysis.

**Table 3:** Gene expression matrix after 30 days rTMS on cerebrum.

**TC ID**	**GS**	**L1**	**C**	**L2**	**C**	**L3**	**C**	**L4**	**C**	*	**#**	**Description**
103611_at	Cd47	0.55	I	0.59	I	-0.22	NC	-0.18	NC	2	0	CD47 antigen (Rh-related antigen, integrin-associated signal
												transducer)
103613_at	Aldoart2	1.82	I	1.52	I	1.42	NC	1.55	NC	2	0	aldolase 1 A, retrogene 2
103624_at	Urm1	0.25	NC	-0.2	NC	0.54	I	0.36	I	2	0	ubiquitin related modifier 1 homolog (S. cerevisiae)
103663_at	Pomgnt1	0.35	I	0.32	I	-0.16	NC	0.07	NC	2	0	protein O-linked mannose beta 1,2-N-acetylglucosaminyltransferase
103682_at	Uri1	-0.04	NC	0.65	I	-0.23	NC	0.27	I	2	0	URI1, prefoldin-like chaperone
103748_at	Cmip	0.78	I	0.34	I	-0.03	NC	-0.27	NC	2	0	c-Maf inducing protein
103771_at	Rnf208	0.48	I	0.17	I	-0.16	NC	-0.32	NC	2	0	ring finger protein 208
104032_at	Mast3	0.6	I	0.36	I	-0.39	NC	-0.51	NC	2	0	microtubule associated serine/threonine kinase 3
104034_at	AI464131	0.63	I	0.66	I	-0.77	NC	-0.4	NC	2	0	expressed sequence AI464131
104214_at	Slc7a8	0.63	I	0.33	I	0.09	NC	0.16	NC	2	0	solute carrier family 7 (cationic amino acid transporter,
												y+ system).member 8
104244_at	Mark2	0.95	I	0.63	I	-0.35	NC	-0.5	NC	2	0	MAP/microtubule affinity regulating kinase 2
104250_at	Lrrc8a	0.53	I	0.12	I	-0.36	NC	-0.64	NC	2	0	leucine rich repeat containing 8A
104316_at	Gna13	0.64	I	0.59	I	-0.02	NC	-0.01	NC	2	0	guanine nucleotide binding protein, alpha 13
104352_at	Brd4	0.2	MI	0.41	I	-0.37	NC	-0.33	NC	2	0	bromodomain containing 4
104368_at	Mapre3	0.23	MI	0.56	I	-0.22	NC	-0.09	NC	2	0	microtubule-associated protein, RP/EB family, member 3
104380_at	Slc35a1	-0.23	NC	0.34	I	0.02	NC	0.7	I	2	0	solute carrier family 35 (CMP-sialic acid transporter), member 1
104409_at	Grik5	0.49	I	0.37	I	-0.16	NC	-0.25	NC	2	0	glutamate receptor, ionotropic, kainate 5 (gamma 2)
104415_at	Foxp1	0.47	I	0.2	I	-0.12	NC	-0.18	NC	2	0	forkhead box P1
104514_at	Epn1	0.3	I	0.11	I	-0.17	NC	-0.39	NC	2	0	epsin 1
104546_g_at	Csnk2a1	1.1	I	-0.1	NC	0.97	I	0.13	NC	2	0	casein kinase 2, alpha 1 polypeptide; predicted pseudogene 10031
104634_at	Lims1	0.49	NC	0.73	I	-0.16	NC	0.5	I	2	0	LIM and senescent cell antigen-like domains 1
104650_at	Ache	0.48	I	0.38	I	0.19	NC	-0.12	NC	2	0	acetylcholinesterase
104725_at	Rhoq	0.81	I	0.61	I	0.37	NC	0.02	NC	2	0	ras homolog gene family, member Q
104739_at	Tcta	0.18	NC	-0.34	NC	0.66	I	0.31	I	2	0	T cell leukemia translocation altered gene
104741_at	Zdhhc9	0.8	I	0.14	I	0.75	NC	-0.1	NC	2	0	zinc finger, DHHC domain containing 9
104747_at	Slc1a1	0.42	I	0.44	I	-0.28	NC	-0.35	NC	2	0	solute carrier family 1 (neuronal/epithelial high affinity glutamate
												transporter, system Xag), member 1
160111_at	Eif1ax	-0.38	NC	-0.47	NC	0.48	I	0.31	I	2	0	eukaryotic translation initiation factor 1A, X-linked
160181_at	Syp	0.37	I	0.45	I	-0.43	NC	-0.42	NC	2	0	synaptophysin
160184_at	Ergic1	0.75	I	0.12	NC	0.75	I	-0.22	NC	2	0	endoplasmic reticulum-golgi intermediate compartment (ERGIC) 1
160190_at	Syt4	0.5	I	0.68	I	-0.09	NC	-0.01	NC	2	0	synaptotagmin IV
160196_at	Smap1	0.37	I	0.42	I	-0.59	NC	-0.48	NC	2	0	small ArfGAP 1
160272_at	Cbx3	0.55	I	0.55	I	-0.41	NC	-0.13	NC	2	0	chromobox 3
160414_at	Slc38a10	0.42	I	-0.17	NC	0.28	I	-0.26	NC	2	0	solute carrier family 38, member 10
160417_at	Kif5b	0.34	I	0.7	I	-0.23	NC	0.08	NC	2	0	kinesin family member 5B
160502_at	Creg1	-0.03	NC	-0.22	NC	0.52	MI	0.14	I	2	0	cellular repressor of E1A-stimulated genes 1
160614_at	Pten	0.6	I	0.39	I	-0.13	NC	-0.36	NC	2	0	phosphatase and tensin homolog
160667_at	Evl	0.58	I	0.56	I	-0.2	NC	-0.34	NC	2	0	Ena-vasodilator stimulated phosphoprotein
160743_at	Pole3	0.41	I	-0.13	NC	0.15	I	-0.44	NC	2	0	polymerase (DNA directed), epsilon 3 (p17 subunit)
160754_at	Pygm	0.66	I	0.83	I	-0.34	NC	-0.1	NC	2	0	muscle glycogen phosphorylase
160942_at	Cbx6	0.63	I	0.23	I	-0.06	NC	-0.36	NC	2	0	chromobox 6
161015_at	Clvs1	0.11	NC	0.7	I	-0.06	NC	0.71	I	2	0	clavesin 1
161054_at	Spock1	0.62	I	0.44	I	-0.13	NC	-0.05	NC	2	0	sparc/osteonectin, cwcv and kazal-like domains proteoglycan 1
161057_at	Actr10	0.35	I	0.33	I	-0.36	NC	-0.41	NC	2	0	ARP10 actin-related protein 10

Ache is Cholinergic gene. Grik5 and Slc1a1 are Glutamatergic gene. Mice cerebrum stimulated by rTMS for 30days were denoted as M1 and M2, sham control were denoted as C1 and C2. N=2. All expression ratios were converted into the log_2_(expression ratio) values. L1: Signal Log Ratio (M1/C1), L2: Signal Log Ratio (M2/C1), L3: Signal Log Ratio (M1/C2), L4: Signal Log Ratio (M1/C2). C1, C2 were used as a control and M1, M2 were normalized with respect to C1 and C2 to obtain expression ratios. Expression Console (EC) Ver.1.4 and Transcriptome Analysis Console (TAC) Ver.3. were used for Comparison Analysis as manufacture procedure. C means data analysis output for a Comparison Analysis illustrating Change p-values with the associated Increase (I) or Decrease (D) call. Increase calls have Change p-values closer to zero and Decrease calls have Change p-values closer to one. Finally, the Change algorithm assesses probe pair saturation, calculates a Change p-values, and assigns an (I), Marginal Increase (MI), No Change (NC), Marginal Decrease (MD), or (D) call for C. Gene with more than 2 significant difference calls was chosen. Abbreviations; TC ID: Transcript Cluster ID, GS: Gene Symbol, *: Total number of increase, #: Total number of decrease. TC ID is available for Pathway analysis.

**Table 4:** Gene expression matrix after 30 days rTMS on cerebrum.

**TC ID**	**GS**	**L1**	**C**	**L2**	**C**	**L3**	**C**	**L4**	**C**	*^​^	**#**	**Description**
161070_at	Spred2	0.28	I	0.38	I	-0.74	NC	-0.5	NC	2	0	sprouty-related, EVH1 domain containing 2
161167_r_at	Uck1	2.05	I	-0.84	NC	2.47	I	-0.66	NC	2	0	uridine-cytidine kinase 1
161371_r_at	Ptprk	1.29	NC	1.29	NC	3.1	I	2.98	I	2	0	protein tyrosine phosphatase, receptor type, K
161819_f_at	Laptm5	0.73	I	0.48	I	-0.11	NC	-0.05	NC	2	0	lysosomal-associated protein transmembrane 5
162134_r_at	2010111I01Rik	0.47	I	-0.14	NC	0.47	I	-0.47	NC	2	0	RIKEN cDNA 2010111I01 gene
162182_f_at	Kcnab2	0.54	I	0.17	I	-0.22	NC	-0.73	NC	2	0	potassium voltage-gated channel, shaker-related subfamily,
												beta member 2
162332_f_at	Mapre3	0.9	I	1.08	I	0.08	NC	-0.03	NC	2	0	microtubule-associated protein, RP/EB family, member 3
162499_f_at	Ube2d2a	0.48	I	0.38	I	-0.17	NC	-0.17	NC	2	0	ubiquitin-conjugating enzyme E2D 2A
92180_at	H1fx	0.42	I	0.37	I	-0.38	NC	-0.51	NC	2	0	H1 histone family, member X
92196_f_at	Sf3a2	0.37	I	0.29	I	-0.16	NC	-0.27	NC	2	0	splicing factor 3a, subunit 2
92202_g_at	Zbtb16	0.72	I	0.66	I	-0.61	NC	-0.73	NC	2	0	zinc finger and BTB domain containing 16
92227_s_at	Ctnna2	0.08	NC	0.36	I	-0.25	NC	-0.01	I	2	0	catenin (cadherin associated protein), alpha 2
92241_at	Nfic	0.68	I	0.88	I	-0.58	NC	-0.43	NC	2	0	nuclear factor I/C
92247_at	Arhgap5	0.6	I	-0.02	NC	0.31	MI	-0.43	NC	2	0	Rho GTPase activating protein 5
92350_at	Mapre1	0.82	I	0.42	I	0.09	NC	-0.44	NC	2	0	microtubule-associated protein, RP/EB family, member 1
92379_f_at	Ptprz1	0.27	MI	0.49	I	-0.55	NC	-0.2	NC	2	0	protein tyrosine phosphatase, receptor type Z, polypeptide 1
92397_at	Agap1	0.52	I	0.2	I	0.06	NC	-0.46	NC	2	0	ArfGAP with GTPase domain, ankyrin repeat and PH domain 1
92411_at	Hs1bp3	0.44	I	0.12	I	0.3	NC	-0.02	NC	2	0	HCLS1 binding protein 3
92426_at	Tspan5	0.33	I	0	NC	0.33	I	-0.14	NC	2	0	tetraspanin 5
92484_at	Hivep2	0.5	I	-0.2	NC	0.36	I	-0.45	NC	2	0	human immunodeficiency virus type I enhancer binding protein 2
92525_i_at	Nacc2	0.33	I	0.82	I	-0.03	NC	0.4	NC	2	0	nucleus accumbens associated 2, BEN and BTB (POZ) domain
												containing
92528_at	Adgrb1	0.37	I	0.17	I	-0.43	NC	-0.55	NC	2	0	adhesion G protein-coupled receptor B1
92586_at	Glud1	0.36	NC	0.39	I	-0.13	NC	0.24	I	2	0	glutamate dehydrogenase 1
92621_at	Pcbp2	0.43	I	0.09	I	-0.08	NC	-0.49	NC	2	0	poly(rC) binding protein 2
92659_at	Rapgef4	-0.28	NC	-0.73	NC	0.98	I	0.66	I	2	0	Rap guanine nucleotide exchange factor (GEF) 4
92678_at	Ddx25	0.22	NC	-0.07	NC	0.17	I	0.12	I	2	0	DEAD (Asp-Glu-Ala-Asp) box polypeptide 25
92795_at	Map4	0.56	I	0.5	I	-0.07	NC	-0.28	NC	2	0	microtubule-associated protein 4
92817_at	Imp3	-0.22	NC	-0.46	NC	0.23	I	-0.03	MI	2	0	IMP3, U3 small nucleolar ribonucleoprotein, homolog (yeast)
92820_at	Usp2	0.64	I	0.54	I	-0.28	NC	-0.34	NC	2	0	ubiquitin specific peptidase 2
92821_at	Usp2	0.42	I	0.29	I	-0.25	NC	-0.27	NC	2	0	ubiquitin specific peptidase 2
92838_at	Fscn1	0.73	I	0.2	NC	0.35	I	-0.16	NC	2	0	fascin homolog 1, actin bundling protein (Strongylocentrotus
												purpuratus)
92871_at	Sel1l	0.79	I	0.4	I	-0.19	NC	-0.36	NC	2	0	sel-1 suppressor of lin-12-like (C. elegans)
92927_at	Etv1	0.66	I	0.27	NC	0.49	I	0.26	NC	2	0	ets variant 1
92949_at	Pacsin1	0.31	I	0.33	I	-0.23	NC	-0.29	NC	2	0	protein kinase C and casein kinase substrate in neurons 1
92958_at	Foxo3	0.43	I	0.11	I	-0.44	NC	-0.54	NC	2	0	forkhead box O3
93006_at	Nfic	0.33	I	0.35	MI	-0.39	NC	-0.58	NC	2	0	nuclear factor I/C
93047_at	Nup50	0.48	I	-0.61	NC	0.81	I	-0.38	NC	2	0	nucleoporin 50
93055_at	Ankrd46	-0.16	NC	-0.14	NC	0.28	I	0.28	I	2	0	ankyrin repeat domain 46
93058_at	Eif1a	0.02	NC	0.35	I	-0.04	NC	0.26	I	2	0	eukaryotic translation initiation factor 1A
93069_at	Ube2d2a	0.04	NC	0.41	I	-0.04	NC	0.2	I	2	0	ubiquitin-conjugating enzyme E2D 2A
93129_at	Cux2	0.51	I	0.43	I	0	NC	-0.28	NC	2	0	cut-like homeobox 2
93147_f_at	Celf4	0.43	I	0.23	I	-0.2	NC	-0.39	NC	2	0	CUGBP, Elav-like family member 4
93246_at	Naa15	0.22	I	0.55	I	-0.27	NC	-0.35	NC	2	0	N(alpha)-acetyltransferase 15, NatA auxiliary subunit
93284_at	Cirbp	-0.27	NC	0.42	I	-0.02	NC	0.54	I	2	0	cold inducible RNA binding protein
93288_at	Arpc2	0.23	MI	0.44	I	-0.31	NC	-0.01	NC	2	0	actin related protein 2/3 complex, subunit 2

Kcnab2 and Pacsin1 are Synaptosome genes. Rapgef4, Arhgap5 and Ctnna2 are Leukocyte transendothelial migration genes. Mice cerebrum stimulated by rTMS for 30days were denoted as M1 and M2, sham control were denoted as C1 and C2. N=2. All expression ratios were converted into the log_2_(expression ratio) values. L1: Signal Log Ratio (M1/C1), L2: Signal Log Ratio (M2/C1), L3: Signal Log Ratio (M1/C2), L4: Signal Log Ratio (M1/C2). C1, C2 were used as a control and M1, M2 were normalized with respect to C1 and C2 to obtain expression ratios. Expression Console (EC) Ver.1.4 and Transcriptome Analysis Console (TAC) Ver.3. were used for Comparison Analysis as manufacture procedure. C means data analysis output for a Comparison Analysis illustrating Change p-values with the associated Increase (I) or Decrease (D) call. Increase calls have Change p-values closer to zero and Decrease calls have Change p-values closer to one. Finally, the Change algorithm assesses probe pair saturation, calculates a Change p-values, and assigns an (I), Marginal Increase (MI), No Change (NC), Marginal Decrease (MD), or (D) call for C. Gene with more than 2 significant difference calls was chosen. Abbreviations; TC ID: Transcript Cluster ID, GS: Gene Symbol, *: Total number of increase, #: Total number of decrease. TC ID is available for Pathway analysis.

**Table 5:** Gene expression matrix after 30 days rTMS on cerebrum.

**TC ID**	**GS**	**L1**	**C**	**L2**	**C**	**L3**	**C**	**L4**	**C**	^*^​^^	**#**	**Description**
93374_at	Jph3	0.44	I	0.38	I	-0.25	NC	-0.17	NC	2	0	junctophilin 3
93382_at	Pde1b	0.61	I	0.29	I	0.04	NC	-0.27	NC	2	0	phosphodiesterase 1B, Ca2+-calmodulin dependent
93423_at	Ldoc1l	0.51	I	0.31	I	0.21	NC	-0.14	NC	2	0	leucine zipper, down-regulated in cancer 1-like
93548_at	Sec. 61b	-0.12	NC	-0.3	NC	0.29	I	0.11	I	2	0	Sec. 61 beta subunit
93645_at	Rgs7	-0.02	NC	0.53	I	-0.03	NC	0.45	I	2	0	regulator of G protein signaling 7
93659_at	Camk2a	1.66	I	-0.95	NC	1.5	I	-0.55	NC	2	0	calcium/calmodulin-dependent protein kinase II alpha
93664_at	Atp1b2	0.78	I	-0.28	NC	0.76	I	-0.15	NC	2	0	ATPase, Na+/K+ transporting, beta 2 polypeptide
93720_at	Agpat1	0.27	MI	-0.71	NC	0.51	I	-0.23	NC	2	0	1-acylglycerol-3-phosphate O-acyltransferase 1
												(lysophosphatidic acid acyltransferase, alpha)
93793_at	Lasp1	0.43	I	0.28	I	-0.14	NC	-0.24	NC	2	0	LIM and SH3 protein 1
93852_at	Mef2a	0.2	I	0.25	I	-0.04	NC	-0.12	NC	2	0	myocyte enhancer factor 2A
93861_f_at	LOC105247328	0.29	I	-0.33	NC	0.56	I	-0.05	NC	2	0	MLV-related proviral Env polyprotein-like
93964_s_at	Ddx6	0.81	I	0.36	I	0.14	NC	-0.46	NC	2	0	DEAD (Asp-Glu-Ala-Asp) box polypeptide 6
93965_r_at	Ddx6	0.88	I	0.59	I	0.23	NC	-0.17	NC	2	0	DEAD (Asp-Glu-Ala-Asp) box polypeptide 6
93994_at	Chpt1	0.4	I	0.27	I	0.15	NC	0.16	NC	2	0	choline phosphotransferase 1
94057_g_at	Scd1	0.32	I	0.43	I	-0.12	NC	-0.03	NC	2	0	stearoyl-Coenzyme A desaturase 1
94077_f_at	Rpn2	0.49	MI	0.29	I	-0.13	NC	-0.27	NC	2	0	ribophorin II
94194_s_at	Hcn2	0.84	I	0.33	I	-0.23	NC	-0.61	NC	2	0	hyperpolarization-activated, cyclic nucleotide-gated K+ 2
94218_at	Tcp1	0.27	I	0.49	I	-0.05	NC	0.35	NC	2	0	t-complex protein 1
94245_at	Vimp	0.08	NC	0.26	I	0.04	NC	0.09	I	2	0	VCP-interacting membrane protein
94257_at	Rraga	0.08	NC	-0.07	NC	0.47	I	0.17	I	2	0	Ras-related GTP binding A
94335_r_at	Ina	0.45	I	0.14	I	-0.25	NC	-0.63	NC	2	0	internexin neuronal intermediate filament protein, alpha
94336_at	Otub1	0.76	I	0.43	I	0	NC	-0.55	NC	2	0	OTU domain, ubiquitin aldehyde binding 1
94353_at	Eif4ebp2	1.04	MI	-0.37	NC	0.4	I	-0.98	NC	2	0	eukaryotic translation initiation factor 4E binding protein 2
94374_at	Wdr13	0.13	NC	0.03	MI	0.06	NC	0.09	MI	2	0	WD repeat domain 13
94456_at	Set	-0.13	NC	0.77	I	-0.14	NC	0.79	I	2	0	SET nuclear oncogene
94819_f_at	Ccni	0.3	I	0.28	I	-0.4	NC	-0.28	NC	2	0	cyclin I
94832_at	Hnrnph2	0.45	NC	0.95	I	0.21	NC	0.83	I	2	0	heterogeneous nuclear ribonucleoprotein H2
94876_f_at	Gorasp2	0.29	I	0.27	I	-0.35	NC	-0.5	NC	2	0	golgi reassembly stacking protein 2
94986_at	Gng3	0.34	I	0.27	I	-0.13	NC	-0.17	NC	2	0	guanine nucleotide binding protein (G protein), gamma 3
95010_at	Traf3	0.29	I	-0.08	NC	0.31	I	-0.02	NC	2	0	TNF receptor-associated factor 3
95159_at	Gm13552	0.1	NC	0.35	I	-0.15	NC	0.22	I	2	0	predicted gene 13552; mitochondrial ribosomal protein S18B
95397_at	D430019H16Rik	0.45	I	0.28	I	0.04	NC	-0.3	NC	2	0	RIKEN cDNA D430019H16 gene
95432_f_at	Tomm70a	0.01	NC	0.35	I	-0.32	NC	0.13	MI	2	0	translocase of outer mitochondrial membrane
												70 homolog A (yeast)
95447_at	Mdp1	-0.4	NC	-0.23	NC	0.43	MI	0.41	I	2	0	magnesium-dependent phosphatase 1
95468_at	Egln1	0.4	I	0.11	NC	0.26	I	0.1	NC	2	0	egl-9 family hypoxia-inducible factor 1
95530_at	Gtf2a1	0.13	NC	-0.1	NC	0.29	I	0.13	I	2	0	general transcription factor II A, 1
95721_at	Mapkapk2	0.36	I	-0.21	NC	0.6	I	-0.02	NC	2	0	MAP kinase-activated protein kinase 2
95883_at	Jade1	0.25	MI	0.54	I	-0.54	NC	-0.14	NC	2	0	jade family PHD finger 1
95927_f_at		0.55	MI	0.65	I	-0.2	NC	-0.14	NC	2	0	
96007_at	Ssr3	-0.12	NC	-0.3	NC	0.24	I	0.16	I	2	0	signal sequence receptor, gamma
96056_at	Rhoc	0.57	I	-0.23	NC	0.46	I	-0.12	NC	2	0	ras homolog gene family, member C
96065_at	Lxn	0.55	I	0.73	I	-0.43	NC	0.12	NC	2	0	latexin
96088_at	Ndrg2	0.44	I	0.48	I	-0.28	NC	-0.34	NC	2	0	N-myc downstream regulated gene 2
96102_i_at	Rad23b	-0.07	NC	0.26	I	-0.26	NC	0.12	I	2	0	RAD23b homolog (S. cerevisiae)
96186_at	Lrp10	0.58	I	-0.1	NC	0.42	I	-0.28	NC	2	0	low-density lipoprotein receptor-related protein 10

Rad23b, Sec. 61b, Vimp, Rpn2 and Ssr3 are genes of protein processing in endoplasmic reticulum. Atp1b2 and Camk2a are cAMP signaling pathway genes. Mice cerebrum stimulated by rTMS for 30days were denoted as M1 and M2, sham control were denoted as C1 and C2. N=2. All expression ratios were converted into the log_2_(expression ratio) values. L1: Signal Log Ratio (M1/C1), L2: Signal Log Ratio (M2/C1), L3: Signal Log Ratio (M1/C2), L4: Signal Log Ratio (M1/C2). C1, C2 were used as a control and M1, M2 were normalized with respect to C1 and C2 to obtain expression ratios. Expression Console (EC) Ver.1.4 and Transcriptome Analysis Console (TAC) Ver.3. were used for Comparison Analysis as manufacture procedure. C means data analysis output for a Comparison Analysis illustrating Change p-values with the associated Increase (I) or Decrease (D) call. Increase calls have Change p-values closer to zero and Decrease calls have Change p-values closer to one. Finally, the Change algorithm assesses probe pair saturation, calculates a Change p-values, and assigns an (I), Marginal Increase (MI), No Change (NC), Marginal Decrease (MD), or (D) call for C. Gene with more than 2 significant difference calls was chosen. Abbreviations; TC ID: Transcript Cluster ID, GS: Gene Symbol, *: Total number of increase, #: Total number of decrease. TC ID is available for Pathway analysis.

**Table 6:** Gene expression matrix after 30 days rTMS on cerebrum.

**TC ID**	**GS**	**L1**	**C**	**L2**	**C**	**L3**	**C**	**L4**	**C**	*^​^	**#**	**Description**
96191_at	Arfgef1	0.59	I	0.17	I	0.24	NC	-0.28	NC	2	0	ADP-ribosylation factor guanine nucleotide-exchange factor 1
												(brefeldin A-inhibited)
96211_at	Dpp8	0.38	I	-0.08	NC	0.23	MI	-0.23	NC	2	0	dipeptidylpeptidase 8
96255_at	Bnip3l	-0.01	NC	0.37	I	-0.09	NC	0.15	I	2	0	BCL2/adenovirus E1B interacting protein 3-like
96313_at	Rasgrf1	0.71	I	0.76	I	-0.25	NC	-0.19	NC	2	0	RAS protein-specific guanine nucleotide-releasing factor 1
96360_at	Arhgdia	0.33	I	0.13	I	-0.02	NC	-0.17	NC	2	0	Rho GDP dissociation inhibitor (GDI) alpha
96518_at	Wwc1	0.33	I	0.31	I	-0.34	NC	-0.5	NC	2	0	WW, C2 and coiled-coil domain containing 1
96593_at	Elk1	0.74	I	-0.53	NC	1.15	I	0.06	NC	2	0	ELK1, member of ETS oncogene family
96674_at	Tnpo3	-0.17	NC	0.57	I	-0.38	NC	0.14	I	2	0	transportin 3
96725_at	Cic	0.81	I	0.27	I	0.12	NC	-0.54	NC	2	0	capicua homolog (Drosophila)
96731_at	Ddx6	0.39	I	0.38	I	-0.09	NC	-0.34	NC	2	0	DEAD (Asp-Glu-Ala-Asp) box polypeptide 6
96741_at	Phf12	0.46	I	0.48	I	-0.46	NC	-0.17	NC	2	0	PHD finger protein 12
96784_at	Anln	-0.27	NC	-0.1	NC	0.7	I	0.32	I	2	0	anillin, actin binding protein
96811_at	Rab31	-0.01	NC	0.01	NC	0.16	I	0.04	I	2	0	RAB31, member RAS oncogene family
96813_f_at	Otud5	0.35	I	0.53	I	-0.33	NC	-0.1	NC	2	0	OTU domain containing 5
96884_at	Carhsp1	-0.1	NC	0.06	NC	0.49	I	0.51	I	2	0	calcium regulated heat stable protein 1
96920_at	Htra1	0.68	I	0.29	I	-0.24	NC	-0.62	NC	2	0	HtrA serine peptidase 1
96955_at	Atp6v0e2	0.04	NC	0.03	NC	0.17	I	-0.01	I	2	0	ATPase, H+ transporting, lysosomal V0 subunit E2
97210_at	1700037H04Rik	0.76	I	-0.13	NC	0.32	I	-0.16	NC	2	0	RIKEN cDNA 1700037H04 gene
97243_at	Slc9a3r1	0.51	I	0.39	I	-0.18	NC	-0.36	NC	2	0	solute carrier family 9 (sodium/hydrogen exchanger), member 3
												regulator 1
97365_at	Coro2b	0.46	I	0.26	I	-0.17	NC	-0.36	NC	2	0	coronin, actin binding protein, 2B
97450_s_at	Aldh7a1	0.46	I	-0.07	NC	0.42	I	-0.22	NC	2	0	aldehyde dehydrogenase family 7, member A1
97487_at	Serpine2	0.32	NC	0.29	I	0.05	NC	0.1	I	2	0	serine (or cysteine) peptidase inhibitor, clade E, member 2
97530_at	Ube2i	0.01	NC	0.18	NC	0.33	I	0.36	I	2	0	ubiquitin-conjugating enzyme E2I
97536_at	Wdtc1	0.68	I	0.03	I	-0.03	NC	-0.49	NC	2	0	WD and tetratricopeptide repeats 1
97740_at	Dusp16	0.46	NC	0.53	I	0.46	NC	0.69	MI	2	0	dual specificity phosphatase 16
97770_s_at	Fam3c	0.53	I	0.63	I	-0.43	NC	-0.49	NC	2	0	family with sequence similarity 3, member C
97776_at	Drd2	0.75	I	1.11	I	0.1	NC	0.27	NC	2	0	dopamine receptor D2
97794_at	Sema7a	2.38	I	-0.83	NC	4.45	I	0.15	NC	2	0	sema domain, immunoglobulin domain (Ig), and GPI membrane
												anchor, (semaphorin) 7A
97841_at	Chmp2a	0.05	NC	0.37	I	-0.18	NC	0.28	I	2	0	charged multivesicular body protein 2A
97974_at	Zfpm1	0.72	I	0.2	I	0.12	NC	-0.54	NC	2	0	zinc finger protein, multitype 1
97998_at	Atn1	0.7	I	0.44	I	0.19	NC	-0.03	NC	2	0	atrophin 1
98004_at	Pkia	0.11	NC	-0.53	NC	1.12	I	0.31	I	2	0	protein kinase inhibitor, alpha
98011_at	Gabbr1	0.24	I	0.3	I	-0.21	NC	-0.23	NC	2	0	gamma-aminobutyric acid (GABA) B receptor, 1
98026_g_at	Evi2a	-0.31	NC	-0.24	NC	0.28	I	0.6	I	2	0	ecotropic viral integration site 2a
98073_at	Cux1	0.46	I	0.27	I	0.24	NC	-0.05	NC	2	0	cut-like homeobox 1
98114_at	Npc1	0.49	I	0.38	I	-0.11	NC	-0.24	NC	2	0	Niemann-Pick type C1
98127_at	Capza2	0.09	NC	0.5	I	-0.11	NC	0.22	I	2	0	capping protein (actin filament) muscle Z-line, alpha 2
98129_at	Tmsb10	0.35	I	0.25	MI	-0.41	NC	-0.72	NC	2	0	thymosin, beta 10
98141_at	Eif5b	0.34	I	0.43	I	-0.58	NC	-0.29	NC	2	0	eukaryotic translation initiation factor 5B
98150_at	Rab11b	0.27	I	0.19	I	-0.23	NC	-0.31	NC	2	0	RAB11B, member RAS oncogene family
98169_s_at	Fzd3	0.39	I	-0.49	NC	0.46	I	-0.41	NC	2	0	frizzled homolog 3 (Drosophila)
98454_at	Palm	0.4	I	0.14	MI	0	NC	-0.25	NC	2	0	paralemmin
98477_s_at	Ank3	0.57	I	0.29	I	-0.29	NC	-0.51	NC	2	0	ankyrin 3, epithelial

Arfgef1, Rab31, Rab11b, Capza2, and Chmp2a are genes of endocytosis. Mice cerebrum stimulated by rTMS for 30days were denoted as M1 and M2, sham control were denoted as C1 and C2. N=2. All expression ratios were converted into the log_2_(expression ratio) values. L1: Signal Log Ratio (M1/C1), L2: Signal Log Ratio (M2/C1), L3: Signal Log Ratio (M1/C2), L4: Signal Log Ratio (M1/C2). C1, C2 were used as a control and M1, M2 were normalized with respect to C1 and C2 to obtain expression ratios. Expression Console (EC) Ver.1.4 and Transcriptome Analysis Console (TAC) Ver.3. were used for Comparison Analysis as manufacture procedure. C means data analysis output for a Comparison Analysis illustrating Change p-values with the associated Increase (I) or Decrease (D) call. Increase calls have Change p-values closer to zero and Decrease calls have Change p-values closer to one. Finally, the Change algorithm assesses probe pair saturation, calculates a Change p-values, and assigns an (I), Marginal Increase (MI), No Change (NC), Marginal Decrease (MD), or (D) call for C. Gene with more than 2 significant difference calls was chosen. Abbreviations; TC ID: Transcript Cluster ID, GS: Gene Symbol, *: Total number of increase, #: Total number of decrease. TC ID is available for Pathway analysis.

**Table 7:** Gene expression matrix after 30 days rTMS on cerebrum.

**TC ID**	**GS**	**L1**	**C**	**L2**	**C**	**L3**	**C**	**L4**	**C**	*	**#**	**Description**
98543_at	Ctss	-0.18	NC	0.3	I	-0.03	NC	0.25	I	2	0	cathepsin S
98550_at	Set	-0.1	NC	0.46	I	-0.25	NC	0.09	I	2	0	SET nuclear oncogene
98564_f_at	Gm6654	0.39	I	0.47	I	-0.29	NC	-0.2	NC	2	0	predicted pseudogene 6654; 40S ribosomal protein S26-like;
												ribosomal protein S26
98588_at	Fah	-0.15	NC	-0.21	NC	0.21	I	0.58	I	2	0	fumarylacetoacetate hydrolase
98590_at	Sdc4	0.69	I	0.12	I	-0.42	NC	-0.76	NC	2	0	syndecan 4
98602_at	Rangap1	0.5	I	0.27	I	0.09	NC	-0.01	NC	2	0	RAN GTPase activating protein 1
98616_f_at	Myh7	0.64	I	0.16	I	-0.43	NC	-1.01	NC	2	0	myosin, heavy polypeptide 7, cardiac muscle, beta
98827_i_at	Kif5a	0.24	NC	-1.04	NC	1.74	I	0.33	I	2	0	kinesin family member 5 A
98866_at	Dlx6	0.92	I	1.99	I	0.7	NC	2.14	NC	2	0	distal-less homeobox 6
98925_at	Vamp2	0.91	I	0.33	NC	0.76	I	0.19	NC	2	0	vesicle-associated membrane protein 2
98993_at	Ppp2r5c	0.23	MI	0.35	I	0.08	NC	0.02	NC	2	0	protein phosphatase 2, regulatory subunit B', gamma
99023_at	Pafah1b2	-0.09	NC	-0.42	NC	0.66	I	0.26	I	2	0	platelet-activating factor acetylhydrolase, isoform 1b, subunit 2
99045_at	Eno2	0.36	I	0.34	I	-0.38	NC	-0.49	NC	2	0	enolase 2, gamma neuronal
99085_at	Usp3	0.41	NC	0.22	NC	0.65	I	0.4	I	2	0	ubiquitin specific peptidase 3
99378_f_at	H2-Q4	-0.42	NC	-0.42	NC	0.61	MI	0.53	I	2	0	histocompatibility 2, Q region locus 4
99451_at	Fam102a	0.36	I	0.16	NC	0.62	I	0.11	NC	2	0	family with sequence similarity 102, member A
99465_at	Mecp2	0.69	I	0.59	I	0.08	NC	-0.04	NC	2	0	methyl CpG binding protein 2
99504_at	St8sia3	0.38	I	0.2	MI	-0.22	NC	-0.3	NC	2	0	ST8 alpha-N-acetyl-neuraminide alpha-2,8-sialyltransferase 3
99537_at	Ruvbl1	0.03	NC	-0.28	NC	0.48	I	0.1	I	2	0	RuvB-like protein 1
99575_at	Ubqln1	0.52	NC	-0.16	NC	0.6	I	0.13	I	2	0	ubiquilin 1
99597_at	Gnai2	0.45	I	0.23	MI	-0.11	NC	-0.34	NC	2	0	guanine nucleotide binding protein (G protein), alpha inhibiting 2
99666_at	Cs	0.39	I	0.14	I	0.06	NC	-0.21	NC	2	0	citrate synthase
99893_at	Fgf13	0.07	NC	-0.57	NC	0.87	I	0.05	I	2	0	fibroblast growth factor 13

Gnai2, Kif5a and Ppp2r5c are dopaminergic synapse genes. Gnai2, Myh7 and Ppp2r5c are genes of adrenergic signaling in cardiomyocytes. Mice cerebrum stimulated by rTMS for 30days were denoted as M1 and M2, sham control were denoted as C1 and C2. N=2. All expression ratios were converted into the log_2_(expression ratio) values. L1: Signal Log Ratio (M1/C1), L2: Signal Log Ratio (M2/C1), L3: Signal Log Ratio (M1/C2), L4: Signal Log Ratio (M1/C2). C1, C2 were used as a control and M1, M2 were normalized with respect to C1 and C2 to obtain expression ratios. Expression Console (EC) Ver.1.4 and Transcriptome Analysis Console (TAC) Ver.3. were used for Comparison Analysis as manufacture procedure. C means data analysis output for a Comparison Analysis illustrating Change p-values with the associated Increase (I) or Decrease (D) call. Increase calls have Change p-values closer to zero and Decrease calls have Change p-values closer to one. Finally, the Change algorithm assesses probe pair saturation, calculates a Change p-values, and assigns an (I), Marginal Increase (MI), No Change (NC), Marginal Decrease (MD), or (D) call for C. Gene with more than 2 significant difference calls was chosen. Abbreviations; TC ID: Transcript Cluster ID, GS: Gene Symbol, *: Total number of increase, #: Total number of decrease. TC ID is available for Pathway analysis.

**Table 8:** Gene expression matrix after 30 days rTMS on cerebrum.

**TC ID**	**GS**	**L1**	**C**	**L2**	**C**	**L3**	**C**	**L4**	**C**	*	**#**	**Description**
102362_i_at	Junb	-2.26	D	-1.89	D	-1.91	D	-1.6	D	0	4	jun B proto-oncogene
102371_at	Nr4a1	-1.99	D	-1.81	D	-1.8	D	-1.72	D	0	4	nuclear receptor subfamily 4, group A, member 1
102661_at	Egr2	-1.29	D	-1.33	D	-1.7	D	-1.69	D	0	4	early growth response 2
102870_at	Dynlt1a	-0.68	D	-1.04	D	-0.62	D	-0.91	D	0	4	dynein light chain Tctex-type 1A
104598_at	Dusp1	-1.41	D	-1.38	D	-1.51	D	-1.49	D	0	4	dual specificity phosphatase 1
160172_at	Meg3	-1.61	D	-1.73	D	-0.68	D	-0.89	D	0	4	maternally expressed 3
160173_at	Meg3	-0.91	D	-1.31	D	-0.79	D	-1.15	D	0	4	maternally expressed 3
160901_at	Fos	-2.14	D	-2.01	D	-2.08	D	-1.95	D	0	4	FBJ osteosarcoma oncogene
160970_at	Odf2	-0.95	D	-1.24	D	-1.31	D	-1.54	D	0	4	outer dense fiber of sperm tails 2
161666_f_at	Gadd45b	-0.98	D	-0.99	D	-1.63	D	-1.51	D	0	4	growth arrest and DNA-damage-inducible 45 beta
96302_at	Srsf7	-0.83	D	-0.73	D	-0.82	D	-0.74	MD	0	4	serine/arginine-rich splicing factor 7
97752_at	Snhg11	-1.12	D	-0.63	D	-1.24	D	-0.71	D	0	4	small nucleolar RNA host gene 11
97890_at	Sgk1	-0.8	D	-1.04	D	-1.3	D	-1.59	D	0	4	serum/glucocorticoid regulated kinase 1
99109_at	Ier2	-0.92	D	-1.48	D	-0.87	D	-1.29	D	0	4	immediate early response 2
101058_at	Amy1	-1.11	D	-0.83	D	-1.08	MD	-0.81	NC	0	3	amylase 1, salivary
101583_at	Btg2	-0.58	D	-1.06	D	-0.6	NC	-1.04	D	0	3	B cell translocation gene 2, anti-proliferative
104410_at	Midn	-0.72	D	-0.23	NC	-1.07	D	-0.77	MD	0	3	midnolin
104639_i_at	Taf1d	-1.01	D	-0.74	D	-0.55	D	-0.63	NC	0	3	TATA box binding protein (Tbp)-associated factor,
												RNA polymerase I, D
160487_at	Myl4	-0.35	MD	-0.38	NC	-1.02	D	-0.96	D	0	3	myosin, light polypeptide 4
92424_at	Zfp692	-0.86	D	-0.94	D	-0.42	NC	-0.9	D	0	3	zinc finger protein 692
92542_at	Rsrp1	-1.25	D	-0.75	D	-0.96	D	-0.52	NC	0	3	arginine/serine rich protein 1
93411_at	Sema7a	-0.51	D	-0.1	NC	-1.37	D	-0.75	D	0	3	sema domain, immunoglobulin domain (Ig), and GPI membrane
												anchor, (semaphorin) 7A
93619_at	Per1	-0.9	D	-0.3	NC	-1.01	D	-0.8	D	0	3	period circadian clock 1
98579_at	Egr1	-0.68	D	-0.57	NC	-1.42	D	-1.04	D	0	3	early growth response 1
99347_f_at	Eml5	-0.92	D	-1.48	D	-0.97	NC	-1.44	D	0	3	echinoderm microtubule associated protein like 5
100002_at	Itih3	0.54	I	0.22	NC	-0.65	D	-0.82	D	1	2	inter-alpha trypsin inhibitor, heavy chain 3
100592_at	Ghitm	-0.56	D	-1.06	D	0.52	I	-0.01	NC	1	2	growth hormone inducible transmembrane protein
100599_at	Atf4	-0.82	D	-0.58	D	-0.27	NC	-0.01	MI	1	2	activating transcription factor 4
162457_f_at	Hba-a1	0.7	I	-1.46	D	-0.12	NC	-2.33	D	1	2	hemoglobin alpha, adult chain 1; hemoglobin alpha, adult chain 2
93722_at	Ensa	-0.46	MD	-0.83	D	0.38	I	-0.03	NC	1	2	endosulfine alpha
93909_f_at	Noct	-0.61	D	-0.84	D	0.35	I	0.08	NC	1	2	nocturnin
94781_at	Hba-a1	0.35	I	-1.71	D	-0.38	NC	-2.42	D	1	2	hemoglobin alpha, adult chain 1
97263_s_at	Csnk1d	-0.65	D	-1.23	D	0.13	I	-0.4	NC	1	2	casein kinase 1, delta
99009_at	Nnt	0.77	I	-0.6	D	-0.1	NC	-1.56	D	1	2	nicotinamide nucleotide transhydrogenase
99095_at	Max	-0.62	D	0.36	NC	-0.98	D	0.01	I	1	2	Max protein
100050_at	Id1	-0.75	D	-0.55	NC	-0.64	D	-0.49	NC	0	2	inhibitor of DNA binding 1
100064_f_at	Gja1	0.23	NC	-0.09	NC	-0.62	D	-0.7	D	0	2	gap junction protein, alpha 1
100348_at	Gm40022	-0.82	D	-0.93	MD	-1.22	NC	-1.12	NC	0	2	predicted gene, 40022
100482_at	Zfp598	-0.42	D	-0.81	D	-0.33	NC	-0.64	NC	0	2	zinc finger protein 598
100536_at	Mobp	-0.11	NC	-0.22	NC	-0.72	D	-0.66	D	0	2	myelin-associated oligodendrocytic basic protein
100611_at	Lyz2	0.32	NC	-1.04	D	0.06	NC	-1.05	D	0	2	lysozyme 2
101482_at	Ppp1cc	0.08	NC	-0.04	NC	-0.59	D	-0.77	D	0	2	protein phosphatase 1, catalytic subunit, gamma isoform
101580_at	Cox7b	-0.62	D	-0.43	NC	-0.71	D	-0.43	NC	0	2	cytochrome c oxidase subunit VIIb
101596_at	C78859	-0.96	MD	-0.8	MD	-0.66	NC	-0.91	NC	0	2	expressed sequence C78859
101740_at	Adra1a	-0.04	NC	-2.3	D	-1.47	NC	-4.05	D	0	2	adrenergic receptor, alpha 1a

Atf4, Adra1a, Myl4 and Ppp1cc are genes of adrenergic signaling in cardiomyocytes. Fos, Max, Atf4, Dusp1, Gadd45b and Nr4a1 are genes of MAPK signaling pathway. Fos, Atf4 and Ppp1cc are Dopaminergic genes. Csnk1d, Id1 and Ppp1cc are genes of hippo signaling pathway. Csnk1d and Per1 are circadian rhythm genes. Mice cerebrum stimulated by rTMS for 30days were denoted as M1 and M2, sham control were denoted as C1 and C2. N=2. All expression ratios were converted into the log_2_(expression ratio) values. L1: Signal Log Ratio (M1/C1), L2: Signal Log Ratio (M2/C1), L3: Signal Log Ratio (M1/C2), L4: Signal Log Ratio (M1/C2). C1, C2 were used as a control and M1, M2 were normalized with respect to C1 and C2 to obtain expression ratios. Expression Console (EC) Ver.1.4 and Transcriptome Analysis Console (TAC) Ver.3. were used for Comparison Analysis as manufacture procedure. C means data analysis output for a Comparison Analysis illustrating Change p-values with the associated Increase (I) or Decrease (D) call. Increase calls have Change p-values closer to zero and Decrease calls have Change p-values closer to one. Finally, the Change algorithm assesses probe pair saturation, calculates a Change p-values, and assigns an (I), Marginal Increase (MI), No Change (NC), Marginal Decrease (MD), or (D) call for C. Gene with more than 2 significant difference calls was chosen. Abbreviations; TC ID: Transcript Cluster ID, GS: Gene Symbol, *: Total number of increase, #: Total number of decrease. TC ID is available for Pathway analysis.

**Table 9:** Gene expression matrix after 30 days rTMS on cerebrum.

**TC ID**	**GS**	**L1**	**C**	**L2**	**C**	**L3**	**C**	**L4**	**C**	*	**#**	**Description**
101869_s_at	Hbb-b1	0.03	NC	-1.76	D	-0.05	NC	-1.94	D	0	2	hemoglobin, beta adult major chain; hemoglobin, beta adult minor
												chain; hemoglobin, beta adult s chain; hemoglobin, beta adult t chain
101936_at	Clk4	-0.63	D	-0.73	D	-0.4	NC	-0.3	NC	0	2	CDC like kinase 4
101962_at	Ddx17	-0.73	D	-0.35	NC	-0.58	D	-0.19	NC	0	2	DEAD (Asp-Glu-Ala-Asp) box polypeptide 17
102255_at	Osmr	0.04	NC	-1.08	MD	-3.48	NC	-3.02	MD	0	2	oncostatin M receptor
102431_at	Mapt	-0.01	NC	-0.04	NC	-0.61	D	-0.6	D	0	2	microtubule-associated protein tau
102574_at	Fgf11	2.96	NC	-1.26	D	3.35	NC	-0.77	D	0	2	fibroblast growth factor 11
102779_at	Gadd45b	-1.04	D	-1.15	D	-0.64	NC	-0.62	NC	0	2	growth arrest and DNA-damage-inducible 45 beta
102781_at	Ccnl2	-0.78	D	-0.93	D	-0.72	NC	-0.79	NC	0	2	cyclin L2
103253_at	Lin7b	-0.46	D	-0.39	NC	-0.64	D	-0.56	NC	0	2	lin-7 homolog B (C. elegans)
103427_at	Fbxl3	-0.67	D	-0.63	D	-0.2	NC	-0.39	NC	0	2	F-box and leucine-rich repeat protein 3
103448_at	S100a8	2.13	NC	-2.11	D	1.42	NC	-2.74	D	0	2	S100 calcium binding protein A8 (calgranulin A)
103460_at	Ddit4	-0.34	NC	-0.5	NC	-1.1	D	-1.48	D	0	2	DNA-damage-inducible transcript 4
103534_at	Hbb-b2	0.17	NC	-1.63	D	-0.09	NC	-2.34	D	0	2	hemoglobin, beta adult minor chain
103811_at	Invs	-0.58	NC	-1.39	D	-1.34	NC	-2	D	0	2	inversin
103863_at	Sft2d1	-0.41	D	-0.66	D	-0.18	NC	-0.29	NC	0	2	SFT2 domain containing 1
103990_at	Fosb	0.08	NC	-1.24	D	-0.3	NC	-1.35	D	0	2	FBJ osteosarcoma oncogene B
104155_f_at	Atf3	-1.59	D	-2.13	D	-0.47	NC	-1.05	NC	0	2	activating transcription factor 3
104578_f_at	Actn1	0.08	NC	-0.05	NC	-0.69	D	-0.86	D	0	2	actinin, alpha 1
104640_f_at	Taf1d	-0.02	NC	-0.47	NC	-0.68	D	-0.97	D	0	2	TATA box binding protein (Tbp)-associated factor,
												RNA polymerase I, D
104701_at	Bhlhe40	-0.38	NC	-0.42	NC	-0.87	D	-0.9	D	0	2	basic helix-loop-helix family, member e40
160140_at	Tbce	-1.09	D	-0.66	NC	-0.92	D	-0.82	NC	0	2	tubulin-specific chaperone E
160182_at	Srsf6	-0.6	D	-0.7	D	-0.45	NC	-0.56	NC	0	2	serine/arginine-rich splicing factor 6
160316_at	AI503316	-0.89	D	-1.11	D	-0.01	NC	-0.74	NC	0	2	expressed sequence AI503316; heterogeneous nuclear
												ribonucleoprotein U
160407_at	Actr1a	-1.42	D	-2.18	D	-0.11	NC	-0.68	NC	0	2	ARP1 actin-related protein 1 A, centractin alpha
160547_s_at	Txnip	-0.6	NC	-1.07	D	-0.7	NC	-1.28	D	0	2	thioredoxin interacting protein
160564_at	Lcn2	-0.8	NC	-3.16	D	-0.92	NC	-3.84	D	0	2	lipocalin 2
160791_at	Luc7l3	-0.9	D	-0.14	NC	-1.26	D	-0.37	NC	0	2	LUC7-like 3 (S. cerevisiae)
160894_at	Cebpd	-0.15	NC	-1.06	D	-0.34	NC	-1.37	D	0	2	CCAAT/enhancer binding protein (C/EBP), delta
161462_r_at	6820431F20Rik	-1.44	NC	-2.05	D	-1.53	NC	-2.36	D	0	2	cadherin 11 pseudogene; predicted gene, 21811
162093_at	Yars	-0.87	D	-0.94	D	-0.07	NC	-0.35	NC	0	2	tyrosyl-tRNA synthetase
162399_f_at	Atxn2	-0.14	NC	-0.23	NC	-0.94	D	-1.23	D	0	2	ataxin 2
162424_f_at	Ddx17	-0.58	D	-0.11	NC	-0.86	D	-0.23	NC	0	2	DEAD (Asp-Glu-Ala-Asp) box polypeptide 17
162459_f_at	Col6a1	-0.11	NC	-0.78	NC	-3.33	D	-4.01	D	0	2	collagen, type VI, alpha 1
92211_at	Bod1l	-0.56	D	-0.69	D	-0.56	NC	-0.5	NC	0	2	biorientation of chromosomes in cell division 1-like
92233_at	Paxbp1	-0.62	D	-0.93	D	-0.61	NC	-0.68	NC	0	2	PAX3 and PAX7 binding protein 1
92523_at	Kcnj6	0.42	NC	-1.73	D	1.6	NC	-1.02	MD	0	2	potassium inwardly-rectifying channel, subfamily J, member 6
92636_f_at	Gm10177	-0.34	D	-0.09	NC	-0.52	D	-0.23	NC	0	2	predicted gene 10177; predicted gene, 17756; predicted gene 4184;
												protein transport protein Sec. 61 subunit gamma; protein transport
												protein Sec. 61 subunit gamma-like; SEC. 61, gamma subunit
92642_at	Car2	-0.42	MD	-0.37	NC	-0.88	D	-0.72	NC	0	2	carbonic anhydrase 2
92665_f_at	3830403N18Rik	-3.41	D	-3.56	NC	-3.93	D	-4.21	NC	0	2	RIKEN cDNA 3830403N18 gene; X-linked lymphocyte-regulated
92751_i_at	Wnt10b	0.49	NC	-1.75	D	-2.13	NC	-4.2	D	0	2	wingless-type MMTV integration site family, member 10B
92768_s_at	Alas2	-0.01	NC	-1.89	D	-0.66	NC	-2.71	D	0	2	aminolevulinic acid synthase 2, erythroid

Ddit4, Col6a1, Fgf11 and Osmr genes of PI3K-AKT signaling pathway. Fbxl3 and Bhlhe40 are circadian rhythm genes. Mice cerebrum stimulated by rTMS for 30days were denoted as M1 and M2, sham control were denoted as C1 and C2. N=2. All expression ratios were converted into the log_2_(expression ratio) values. L1: Signal Log Ratio (M1/C1), L2: Signal Log Ratio (M2/C1), L3: Signal Log Ratio (M1/C2), L4: Signal Log Ratio (M1/C2). C1, C2 were used as a control and M1, M2 were normalized with respect to C1 and C2 to obtain expression ratios. Expression Console (EC) Ver.1.4 and Transcriptome Analysis Console (TAC) Ver.3. were used for Comparison Analysis as manufacture procedure. C means data analysis output for a Comparison Analysis illustrating Change p-values with the associated Increase (I) or Decrease (D) call. Increase calls have Change p-values closer to zero and Decrease calls have Change p-values closer to one. Finally, the Change algorithm assesses probe pair saturation, calculates a Change p-values, and assigns an (I), Marginal Increase (MI), No Change (NC), Marginal Decrease (MD), or (D) call for C. Gene with more than 2 significant difference calls was chosen. Abbreviations; TC ID: Transcript Cluster ID, GS: Gene Symbol, *: Total number of increase, #: Total number of decrease. TC ID is available for Pathway analysis.

**Table 10:** Gene expression matrix after 30 days rTMS on cerebrum.

**TC ID**	**GS**	**L1**	**C**	**L2**	**C**	**L3**	**C**	**L4**	**C**	*	**#**	**Description**
92777_at	Cyr61	-1.09	NC	-1.87	D	-1.21	NC	-2.36	D	0	2	cysteine rich protein 61
92787_at	Ankrd10	-0.66	D	-1	D	-0.31	NC	-0.69	NC	0	2	ankyrin repeat domain 10
92802_s_at	Plp1	-0.61	D	-0.99	D	0.03	NC	-0.42	NC	0	2	proteolipid protein (myelin) 1
92945_at	Gria2	-0.45	NC	-0.06	NC	-0.96	D	-0.82	D	0	2	glutamate receptor, ionotropic, AMPA2 (alpha 2)
93009_at	Gstm2	-2	D	-4.19	D	-0.85	NC	-2.44	NC	0	2	glutathione S-transferase, mu 2
93126_at	Ckb	-0.01	NC	-0.12	NC	-0.53	D	-0.56	D	0	2	creatine kinase, brain
93274_at	Clk1	-1.11	D	-1.04	D	-0.6	NC	-0.46	NC	0	2	CDC-like kinase 1
93285_at	Dusp6	-0.48	NC	-0.52	NC	-0.58	D	-0.83	D	0	2	dual specificity phosphatase 6
93353_at	Lum	-0.93	D	-0.76	D	0.22	NC	0.25	NC	0	2	lumican
93390_g_at	Prom1	0.11	NC	0.05	NC	-1.44	D	-1.09	D	0	2	prominin 1
93478_at	Dnajc21	-0.14	NC	-0.07	NC	-1.02	MD	-1.39	D	0	2	DnaJ (Hsp40) homolog, subfamily C, member 21
93486_at	Slc27a1	0.53	NC	-0.65	D	-0.4	NC	-1.46	D	0	2	solute carrier family 27 (fatty acid transporter), member 1
93615_at	Pbx3	0.26	NC	0.07	NC	-0.63	D	-0.94	D	0	2	pre B cell leukemia homeobox 3
93666_at	Lmo2	-0.51	D	-0.72	D	0.08	NC	-0.23	NC	0	2	LIM domain only 2
93773_f_at	Zranb2	-0.71	D	-0.4	NC	-0.61	D	-0.41	NC	0	2	zinc finger, RAN-binding domain containing 2
93985_at	Tiparp	-1.11	D	-0.46	NC	-0.74	D	-0.79	NC	0	2	TCDD-inducible poly(ADP-ribose) polymerase
94155_at	Rgs4	-0.56	MD	-0.96	D	0.49	NC	-0.31	NC	0	2	regulator of G-protein signaling 4
94192_at	Gdap10	-0.41	NC	-1.15	D	-0.66	NC	-1.24	D	0	2	ganglioside-induced differentiation-associated-protein 10
94349_at	Khdc1b	-2.59	NC	-4.38	D	0.41	NC	-1.57	D	0	2	KH domain containing 1B
94379_at	Kif1b	-1.4	NC	-1.71	D	-1.62	NC	-2.65	D	0	2	kinesin family member 1B
94395_at	Fubp1	-0.48	NC	-0.78	D	-0.99	D	-1.05	NC	0	2	far upstream element (FUSE) binding protein 1
94460_at	Stk38	-0.43	D	-0.79	D	-0.04	NC	-0.37	NC	0	2	serine/threonine kinase 38
94516_f_at	Penk	0.16	NC	0.02	NC	-0.78	D	-0.89	D	0	2	preproenkephalin
94689_at	Gm39971	-0.03	NC	-0.48	NC	-1.5	D	-2	D	0	2	predicted gene, 39971
95001_at	Akap8	-0.88	D	-1	MD	-0.62	NC	-0.84	NC	0	2	A kinase (PRKA) anchor protein 8
95081_at	Exosc8	-1.38	D	-1.39	D	-0.8	NC	-0.95	NC	0	2	exosome component 8
95134_at	Mid1ip1	-0.92	D	-0.8	D	-0.3	NC	-0.2	NC	0	2	Mid1 interacting protein 1 (gastrulation specific G12-like
												(zebrafish))
95339_r_at	Mmp12	-2.02	MD	-4.34	D	-1.71	NC	-4.09	NC	0	2	matrix metallopeptidase 12
95418_at	Rasl11b	0.02	NC	-0.09	NC	-0.76	D	-0.85	D	0	2	RAS-like, family 11, member B
95674_r_at	Basp1	-0.37	D	-0.21	NC	-0.51	D	-0.3	NC	0	2	brain abundant, membrane attached signal protein 1
95694_at	Top1	-0.2	NC	-0.4	NC	-0.56	D	-0.63	D	0	2	topoisomerase (DNA) I
96464_at	Plxnb2	-0.41	NC	-1.64	D	0.19	NC	-0.78	MD	0	2	plexin B2
96672_at	Hopx	-0.45	D	-0.64	D	-0.29	NC	-0.52	NC	0	2	HOP homeobox
96785_at	Kank3	-0.55	D	-0.33	NC	-1.08	D	-0.92	NC	0	2	KN motif and ankyrin repeat domains 3
96912_s_at	Ctla2a	-0.8	D	-1.02	D	-0.38	NC	-0.77	NC	0	2	cytotoxic T lymphocyte-associated protein 2 alpha; cytotoxic
												T lymphocyte-associated protein 2 beta
												
96961_at	Pcgf2	-0.35	NC	-1.18	D	-0.08	NC	-0.74	D	0	2	polycomb group ring finger 2
97142_at	DXErtd242e	-1.93	D	-2.25	D	-2.93	NC	-3.23	NC	0	2	DNA segment, Chr X, ERATO Doi 242, expressed
97358_at	Adgrl1	0.02	NC	0.08	NC	-0.66	D	-0.5	MD	0	2	adhesion G protein-coupled receptor L1
97759_at	Kcnma1	-0.15	NC	-0.06	NC	-0.87	D	-0.65	D	0	2	potassium large conductance calcium-activated channel,
												subfamily M, alpha member 1
98474_r_at	Tnfaip6	-1.14	D	-0.3	NC	-1.1	MD	0.04	NC	0	2	tumor necrosis factor alpha induced protein 6
98475_at	Matn2	-0.17	NC	-0.29	NC	-0.76	D	-1.16	D	0	2	matrilin 2
99089_at	Mal	-0.28	NC	-0.61	NC	-0.92	D	-1.12	D	0	2	myelin and lymphocyte protein, T cell differentiation protein
99622_at	Klf4	-0.81	NC	-0.52	NC	-2.7	D	-2.56	D	0	2	Kruppel-like factor 4 (gut)
99830_at	Kalrn	-0.16	NC	0.04	NC	-0.89	D	-0.69	D	0	2	kalirin, RhoGEF kinase

Lmo2, Pbx3 and Prom1 are genes of transcriptional misregulation in cancer. Mice cerebrum stimulated by rTMS for 30days were denoted as M1 and M2, sham control were denoted as C1 and C2. N=2. All expression ratios were converted into the log_2_(expression ratio) values. L1: Signal Log Ratio (M1/C1), L2: Signal Log Ratio (M2/C1), L3: Signal Log Ratio (M1/C2), L4: Signal Log Ratio (M1/C2). C1, C2 were used as a control and M1, M2 were normalized with respect to C1 and C2 to obtain expression ratios. Expression Console (EC) Ver.1.4 and Transcriptome Analysis Console (TAC) Ver.3. were used for Comparison Analysis as manufacture procedure. C means data analysis output for a Comparison Analysis illustrating Change p-values with the associated Increase (I) or Decrease (D) call. Increase calls have Change p-values closer to zero and Decrease calls have Change p-values closer to one. Finally, the Change algorithm assesses probe pair saturation, calculates a Change p-values, and assigns an (I), Marginal Increase (MI), No Change (NC), Marginal Decrease (MD), or (D) call for C. Gene with more than 2 significant difference calls was chosen. Abbreviations; TC ID: Transcript Cluster ID, GS: Gene Symbol, *: Total number of increase, #: Total number of decrease. TC ID is available for Pathway analysis.

## 2. Experimental design, materials and methods

We carried out a comprehensive analysis of altered gene expression in cerebrum following chronic rTMS using a high-density oligonucleotide array (GeneChip; Affymetrix, Santa Clara, CA, USA. MG_U74Av2 probe array), as described elsewhere [2]. Using the Affymetrix algorithm [3] and multiple analysis comparison software for assessing gene expression differences, mRNAs that increased or decreased in the mouse brain following chronic rTMS relative to levels in the control mouse brain were identified. Pathway analysis was used to identify the significant pathway of the differential genes according to KEGG. And also, Gene Ontology (GO) analysis was applied to analyze the main function of the differentially expressed genes according to the gene ontology, which is the key functional classification of NCBI that can organize genes into hierarchical categories and uncover the gene regulatory network based on biological process and molecular function [4].

Using these data, we indicated that rTMS modulates glutamate, GABA and glycine transporters and regulates ER stress-related genes [1].
